# Ten Years of Inspiration and Impact: The RANZCP Psychiatry Interest Forum

**DOI:** 10.1192/bjo.2024.310

**Published:** 2024-08-01

**Authors:** Sharon McGowan, Sam Dipnall

**Affiliations:** The Royal Australian and New Zealand College of Psychiatrists, Melbourne, Australia

## Abstract

**Aims:**

To illustrate the scale and impact of the Royal Australian and New Zealand College of Psychiatrists’ (RANZCP) Psychiatry Interest Forum (PIF) ten years since its inception.

**Methods:**

Member data from 2013–2023 was analysed alongside recent event and engagement activity survey results, as well as qualitative feedback from medical students and prevocational doctors who took part in PIF engagement activities.

**Results:**

PIF attracts and inspires the next generation of Australian and New Zealand psychiatrists.

It is a stepping stone into the RANZCP Fellowship program, and has a particular focus on increasing interest in rural careers and supporting more First Nations medical students and prevocational doctors into psychiatry.

PIF events, sponsorships, scholarships and information achieves this by:

- providing a starting point for learning and exploring the specialty of psychiatry
- fostering interest in psychiatry among medical students and junior doctors
- creating a safe and enabling environment to explore the specialty, create networks, and build connections
- challenging common misconceptions about psychiatry and reduce associated stigma
- increasing applications to the RANZCP Fellowship program.

Established in September 2013 the program now has over 5,100 members.

In 2023, the program achieved its highest annual number of new members joining to date, with 1,056 medical students and junior doctors choosing to join PIF. That year, 77% of all new trainees that joined the Fellowship pathway were former PIF members.

Survey data from PIF members who took part in the PIF program hosted at the Perth Congress in 2023 demonstrated that:

- 100% reported an increase in psychiatry knowledge following Congress.
- 82% reported their likelihood of pursuing psychiatry had increased following Congress, and 18% reported ‘no change’, as they reported strong certainty prior to the Congress attendance.
- 75% reported that the PIF networking sessions helped clarify misconceptions or stigmas that they previously held about psychiatry following Congress.

The voices of PIF members best illustrates the influence that inspirational experiences like these can have on future career directions:

*‘My favourite part of the PIF Congress was the ability to interact with likeminded PIF peers and psychiatrists and trainees from all over Australia and New Zealand. Another PIF member had said “I feel like I've found my tribe” which is a comment I particularly resonated with.*

**Conclusion:**

Ten years on, PIF continues to expand its reach and impact to increase the pipeline of psychiatry trainees in Australia and Aotearoa New Zealand.

